# Milk Technological Properties as Affected by Including Artichoke By-Products Silages in the Diet of Dairy Goats

**DOI:** 10.3390/foods6120112

**Published:** 2017-12-18

**Authors:** Raquel Muelas, Paula Monllor, Gema Romero, Estrella Sayas-Barberá, Casilda Navarro, José Ramón Díaz, Esther Sendra

**Affiliations:** AgroFood Techonology Department, Escuela Politécnica Superior de Orihuela, Universidad Miguel Hernández de Elche, Ctra. Beniel km 3,2, 03312 Orihuela, Spain; raquel.muelas@umh.es (R.M.); pmonllor@umh.es (P.M.); gemaromero@umh.es (G.R.); estrella.sayas@umh.es (E.S.-B.); casilda.navarro@umh.es (C.N.); jr.diaz@umh.es (J.R.D.)

**Keywords:** milk quality, milk technology, by-products, artichoke, goat

## Abstract

Traditional farming practices include the use of local agricultural by-products in the diet of ruminants. Artichoke harvesting and transformation yield high amounts of by-products that, if properly used, may reduce farming costs and the environmental impact of farming. The present study tests the inclusion of silages from artichoke by-products (plant and outer bracts) in the diet of dairy goats (0%, 12.5% and 25% inclusion) on the technological and sensory properties of milk during a five-month study. Milk composition, color, stability, coagulation and fermentation properties remained unaffected by diet changes. Panelists were not able to differentiate among yogurts obtained from those milks by discriminant triangular sensory tests. Silages of artichoke by-products can be included in isoproteic and isoenergetic diets for dairy goats, up to a 25% (feed dry matter), without negatively affecting milk technological and sensory properties whereas reducing feeding costs.

## 1. Introduction

By-products from agricultural and food processing industries represent a major disposal problem. The moisture of these by-products is a limitation for their storage and companies must manage them quickly to avoid environmental problems, which imply an important additional cost. Such by-products are also promising sources of valuable compounds. They may be used because of their technological or nutritional properties in animal feeding. One possibility to overcome the short shelf life of these by-products would be their incorporation in silage, which may allow preserving them and obtaining an economic benefit, while reducing the price of the ration and favoring the sustainability of both activities. From the environmental point of view, the reduction of wastes and residues, and the enhanced use of local resources as feed may also reduce the carbon footprint of both horticultural and farming practices and strengthen the local economy.

The cultivation of artichoke is concentrated in the countries bordering the Mediterranean basin. The main European, as well as world, producers are Italy and Spain, with Spain occupying the second position in the world, with a production of 234,091 tons in an approximate area of 15,002 hectares [1]. Within Spain, this crop is particularly important in the autonomous communities of the Mediterranean coast, especially in Murcia, with more than 40% of the national production [2]. More than 40% of artichoke production goes for fresh consumption, while 60% to its industrialization. Its production is seasonal and during its processing huge amounts of by-products are generated, as from 60 to 65% of the artichoke, between outer bracts and stems, is not used.

One main use of such by-products has been animal feed, taking advantage of its large nutritive value [3]. Fresh artichoke by-products have been traditionally used for feeding goats in artichoke-producing areas. However, artichoke production is seasonal, leading to two main constrains: (1) they are not available during the entire year, and; (2) not all generated by-products can be used as animal feed. The huge amounts of by-products generated in a short harvest time are highly perishable due to their high water content. For these reasons, it is necessary to evaluate preserved artichoke by-products (plant: post-harvest by-product and outer bracts: artichoke processing by-product), as silages, in balanced diets for dairy goats to replace part of the usual ration and measure their possible impact on the technological properties of the obtained milk.

Several studies in the scientific literature report the use of different agricultural by-products as feed for ruminants. However, most of them mainly focus on their effect of the fatty acid profile of milk. Some relevant studies are on the following: olive cake inclusion on ewe feeding [4]; linseed meal from flaxseed oil extraction on goat feeding [5]; cassava by-products on lactating dairy goats [6]; citrus pulp in diets of dairy animals [7]; soybean hulls on dairy ewes [8]; *Moringa oleifera* leaf on dairy goats [9]; and mixtures of different by-products, such as tomato and olive by-products [10] or mixture of tomato fruits, citrus pulp, brewer’s grain and yeast [11,12] on dairy goats. Only few studies report the effect of diets including agro-industry by-products on milk composition and technological quality [9,12,13,14], and most of them report successful the incorporation of by-products as feed. It is essential to evaluate new by-products or presentation forms as feed sources and provide the scientific basis for their proper inclusion on balanced diets. No previous studies have been found on the effect of the inclusion of artichoke by-products, in silage form, in dairy goats’ diets on the technological properties of milk. The objective of the present study was to tests the effect of artichoke silage incorporation (plant and outer bracts) in the diet of dairy goats (0%, 12.5% and 25% inclusion) on the technological and sensory properties of milk. 

## 2. Materials and Methods 

### 2.1. Animals and Dietary Treatments

Sixty-nine Murciano-Granadina goats (44.7 ± 6.8 kg of body weight), multiparous and in the third month of lactation were selected from the experimental farm of Escuela Politécnica Superior de Orihuela (Universidad Miguel Hernandez de Elche, Alicante, Spain). They were chosen by number of lactation, milk yield and health status of the mammary gland and distributed in three homogeneous groups (*n* = 23 goats per treatment). At the beginning of the experiment, all goats were housed individually in stalls, and fed with a traditional ration (Control diet: alfalfa hay, barley straw oat and a mix of grains) during a pre-experimental period. These three groups of animals were kept constant during all the study (from October 2015 until February 2016), meaning that individuals in each group were the same for both experiments run, as detailed in the timeline in Table 1. 

After having the animals grouped, the inclusion of by-products (plant and outer bracts) in the diet began, leaving a 15-day period of adaptation to the new feed before the first sampling. All the diets were designed in order to be isoenergetic and isoproteic. During the adaptation period and Experiment 1, diets were formulated in order to cover nutrient requirements of goats weighing 50 kg, with a production level of 2.5 kg/day and 6% fat content. Thus, 2.2 kg of dry matter were offered to each goat every day; although they were not fed ad libitum there were always leftovers. Two experiments were run, corresponding to two inclusion levels of the artichoke by-products in the diets. For Experiment 1, three diets were used during seven weeks: a control diet (CD) without the addition of artichoke by-products in silage; a diet incorporating 12.5% artichoke bracts (ABD-1) in silage; and a diet incorporating 12.5% artichoke plant (APD-1) in silage. The first experiment was followed by a sudden increase in the content of by-products in the diet (25%) that was kept for nine weeks. A four-week period of adaptation feeding 25% by-products was left before the first milk sampling for the second experiment. So, in Experiment 2, three diets were used: a control diet without the addition of artichoke by-products in silage; a diet incorporating 25% artichoke bracts (ABD-2) in silage; and a diet incorporating 25% artichoke plant (APD-2) in silage. All samplings for the second experiments took place within the last five weeks of the experiment. Diets were well accepted by animals, leaving a small quantity of refuse food every day. Intake of dry matter was similar in all groups, both in Experiment 1 and 2. Intake values during Experiment 1 were 2.17, 2.08 and 2.12 for control diet in experiment 1 (CD-1), artichoke bracts diet in experiment 1 (ABD-1) and artichoke plant diet in experiment 1 (APD-1), respectively. During Experiment 2, diets were designed for a lower level of production, so goats were offered less amount of food (1.95 kg of dry matter (DM)/day). Thus, the intake level was also reduced during this period, being 1.91 for control diet in experiment 2 (CD-2), 1.84 for artichoke bracts diet in experiment 2 (ABD-2) and 1.86 for artichoke plant diet in experiment 2 (APD-2). Silages were manufactured by Aprovertia SL, by a process actually under application of Industrial Property Rights protection; it consists of microsilages of 300 kg aged for more than 30 days. The chemical composition and energy of the diets (CD-1, ABD-1, APD-1, CD-2, ABD-2 and APD-2) of both experiments is provided in Table 2. 

### 2.2. Milk Sampling

Milk samples were taken weekly from the tank from each diet group (Table 1). Goats were milked at 9:00 a.m. with a low-line milking machine (Gea-Farm Technologies^®^, Bönen, Germany), and two-liter samples were taken from each tank weekly to run all determinations. The milk samples were refrigerated at 4 °C and they were immediately analyzed for chemical composition, technological characteristics (milk ethanol stability, whey drainage ability and acidification ability) and color. For sensory analysis they were previously pasteurized. During the second experiment 6 L of milk from each diet group were collected to prepare yogurts to be used for sensory evaluation.

### 2.3. Chemical Composition

Milk composition was analyzed using near infrared spectrophotometry (FOSS 120T Milko-Scan; Foss Electric, Hillerфd, Denmark); fat, protein, casein, whey protein, lactose, total solids and ash were determined.

### 2.4. Technological Characteristics

Milk ethanol stability (MES) is usually defined as the highest concentration of added aqueous ethanol which does not cause milk coagulation [15]. It is related to the stability of milk proteins to the heat treatment. Mixtures of equal volume of milk samples and freshly made aqueous ethanol solutions (from 40% at 50%, at 0.5 intervals) were placed in a petri dish and were observed for signals of coagulation. Mixtures (milk:ethanol) were made with different concentrations of ethanol until the concentration of added aqueous ethanol which does not cause coagulation of milk was found. The ethanol stability was expressed as % ethanol. The higher the value the better the stability. Two replicate determinations for each ethanol concentration were carried out for each sample.

Whey drainage ability was determined by quantifying the volume of whey removed from curd after centrifugation [16]. To properly evaluate drainage ability, rennet coagulated curds and acid coagulated curds have to be evaluated. Rennet coagulation (enzymatic draining) at pH 6.3 and direct acidification (acid draining) at 4.5 were attained. For both tests, goat milk samples (20 g) adjusted to pH 6.3, by adding 10% lactic acid were placed in centrifugal tubes. For rennet coagulation, 60 µL of renin (Liquid calf rennet 1:15.000, AC chymosin > 90% and AC pepsin < 10%, Laboratorios Arroyo, Santander, Spain) was added to samples, followed by incubation during 1 h at 30 °C. For direct acidification, 2 µL of renin and 0.2 g of glucono delta lactone (GDL; Sigma-Aldrich, St. Louis, MO, United States) were added, followed by incubation for 20 h at 22 °C. After both incubation times the curds were formed, the tubes were centrifuged at 2800× *g* for 30 min at 20 °C. The whey released from each tube was weighed (g). Whey drainage ability (% whey drained) was expressed as the mass ratio of whey/milk. Whey composition (fat, crude protein and total solid) was determined using near infrared spectrophotometry (FOSS 120T Milko-Scan; Foss Electric, Hillerфd, Denmark). Two replicate determinations were carried for each sample.

Acidification ability was evaluated using lyophilized ferments (MA 400 by Danisco; *Lactococcus lactis* subsp. *lactis, Lactococcus lactis* subsp. *cremoris, Lactococcus lactis* subsp. *lactis biovar diacetylactis, Streptococcus thermophilus*) under standard conditions [16]. Aliquots of goat milk (100 mL) were inoculated with 3.6 mL of the pre-activated starter culture and incubated at 30 ° C. A sample of reconstituted skimmed cow’s milk (10% low-heat milk powder) was also inoculated and incubated in order to verify the viability of lactic starters. Dornic acidity and pH were measured at the beginning of the acidification (A0h), 5 h (A5h) and 24 h (A24h) after inoculation. Dornic acidity was determined by titration with commercial Dornic sodium hydroxide, and pH by using a pHmeter (Crison Instruments S.A., Alella, Spain).

### 2.5. Milk Color Determination

Milk color was measured using a Spectrophotometer CR700d (Konica Minolta, Inc., Osaka Japan), using SCI mode, 10° observer and illuminant D65. During measurements a sample holder CM-A514 (Konica Minolta, Japan) and a rectangular cell made of optical glass for high-precision transmittance (CM-A132 Rectangular Disposal Cell 50 × 38, optical path 20 mm, Minolta, Osaka, Japan) were used. 

The milk samples were homogenized for 30 s on a Vortex and placed in the cell for color determination. The CIELAB color coordinates (L*: lightness; a*: redness; b*: yellowness), chroma (C*) and hue (H*) were studied. Reflectance data over 360 nm to 740 nm were also obtained each milk samples. 

### 2.6. Sensory Analyses 

During both experiments, at 15-day intervals, goat milk samples were pasteurized at 80 °C for 30 min and refrigerated to be further evaluated for the presence of off-flavors. Five members of the department, who are frequent consumers of goat milk, evaluated the milk. In addition, in order to conduct a consumer sensory panel, goat milk yogurts were prepared. To 6 L of milk from each group of the second experiment were added 6% sucrose, and the blend was further pasteurized as previously described. Milk was cooled to 45 °C and inoculated with a commercial starter culture for yogurt (MY800 by Danisco, composed of *Streptococcus thermophilus, Lactobacillus delbrueckii* subsp. *Lactis y Lactobacillus delbrueckii* subsp. *bulgaricus*); inoculated milk was distributed in sterile caps (40 mL each) and incubated at 43 °C until pH 4.6 was reached. After 48 h under refrigeration, a consumer sensory panel (60 panelists) run triangular tests to compare the three types of yogurts (three sets of comparisons each: CD-2 vs. ABD-2, CD-2 vs. APD-2, and ABD-2 vs. APD-2, served in balanced order to consumers). All sensory test were run in the Sensory Laboratory of the Agro-Food Technology Department which fulfills ISO requirements for sensory evaluation. A minimum numbers of correct judgments to establish significance at various probability levels were considered to decide on the significance of the results [17].

### 2.7. Statistical Analysis

The data were analyzed using SPSS 21 (SPSS Statistical Software, Inc., Chicago, IL, USA). Each experiment was independently evaluated. Two fixed effects were studied, namely, diet (CD, ABD and APD) and experiment ( Experiment 1 and Experiment 2: initially sampling time within each experiment was considered, and afterwards removed as sampling time did not affect the studied parameters). The Tukey test was used for the multiple comparisons of means. Differences are considerate significant at *p* > 0.05.

## 3. Results and Discussion

Experiments were consecutively run, as previously explained, and so the stage of lactation moved on with sampling weeks. In order avoid the effect of lactation to interfere with diet effect, two strategies were followed: (1) there was always a control diet group, and; (2) results from samplings within the same experiment and diet were statistically analyzed. As a result, no significant differences were detected among the properties of milk from all samplings of the same experiment. The lactation evolution within each experiment did not affect milk composition and properties, and so results are presented and analyzed separately for each experiment, considering only diet effect. 

### 3.1. Chemical Composition

Table 3 shows the chemical composition of milk from experiments 1 and 2. In the first experiment, the incorporation of artichoke by-products in the diet, independently of whether it was outer bracts or plant, did not modify milk composition. In the Experiment 2, differences were found for crude protein, casein and ash, which were lower when outer bracts were used in feed. However, they may not be considered relevant given the tiny differences (under 0.1 units). 

Increasing fiber content in feed is known to enhance fat synthesis [18], and in the present study crude fiber in APD-2 was slightly lower than that of other diets (Table 2). As well, although no significant differences have been reported for fat content (Table 3), the average fat content for APD-2 milk is the lowest reported in the study. Higher inclusions of by-products need to be evaluated in further studies in order to assess if artichoke plant affects milk fat content. The results obtained in the present study reveal that the inclusion of up to 25% artichoke by-products on dairy goat diets does not relevantly affect milk composition, although it slightly modified crude protein, casein and ash, however, we consider those changes irrelevant due to the reduced differences and absence of impact on milk technological properties.

Uneven results have been reported in the scientific literature for the effect of the inclusion of several agricultural by-products on milk composition. The inclusion of artichoke silages up to a 30% in the ewe’s diet did not affect milk composition [19]. Regarding the use of silages from citrus by-products, also in ewe’s diets an increase in fat and non-fat solids has been reported [20]. Studies including by-products, not as silages, in the diet of goats, like [12] tomato fruits, citrus pulp, brewer’s grain and yeast, reported differences in milk composition and found higher contents of protein, casein and total solids than that of milk from goats fed conventional diet. Romero-Huelva et al. [11] included tomato and cucumber by-products on dairy goats diet and only reported changes on lactose content (*p* < 0.05). Studies with the inclusion of soybeans and linseed in Anglo-Nubia diets [21] reported no changes on milk fat, dry matter and lactose. The increased presence of tannins in goats’ diets tended to increase protein content, however, high tannin contents reduced fat content in milk, whereas lactose and total solids were not affected by tannins inclusion [22]. When rosemary was supplemented in goats’ diets a decrease in dry matter and lactose was reported, whereas fat and protein contents were unaffected [23]. In terms of other species, when olive cake is introduced on ewes’ diet [4], protein and casein contents were smaller than in control milk; the authors explained that fact as due to a dilution effect linked to the higher milk yield on olive cake fed ewes [4]. Most of this studies report slight or none compositional changes, also depending on the percentage of diet replacement by by-products, if few compositional changes are observed, also few changes in technological properties are to be expected, however, information on technological properties is still needed in order to properly assess the suitability of the by-products on dairy goat diets.

### 3.2. Technological Characteristics

Milk ethanol stability. As can be seen in Table 4, no significant differences were observed among milk types for alcohol stability, and for all samplings goats’ milk stand ethanol concentrations over 45% without signs of coagulation, so proving a good stability of milk during both experiments. 

According to Raynal-Lujtovac et al. [15], goat milk tends to destabilize at an ethanol concentration of 45%, whereas cow’s milk stands up to 70% ethanol, and they explain such difference as being due to the different micellar structure of goats and cow’s milk, as well as to the highest ionic calcium in goats milk as compared to cow’s milk. The tested inclusion of artichoke by-product silages did not affect milk ethanol stability.

Acidification capacity. As explained in materials and methods section, reconstituted skimmed cow’s milk was used to check the proper function of commercial cultures each sampling day. Starter cultures performed as expected from culture technical sheet at all sampling days. In Table 4 pH and acidity at 0, 5 and 24 h of incubation are reported and lactic cultures performed in goat milk, as well as they did in control cow’s milk. No significant differences could be attributed to diet, and so the inclusion of up to 25% silages of artichoke by products did not affect milk acidification ability: it had no effect on the growth of lactic acid bacteria on milk. It can be assumed that milk microbial ecology was not affected by the evaluated diets. 

Although other factors may produce effects, it has been reported that protein content may enhance acidification capacity by increasing buffering capacity [16]; no differences in protein content were observed in the present study, nor differences on acidification capacity either.

Whey drainage ability. Results from acid gels wheying off are presented in Table 5, and those from enzymatic gels are in Table 6. The percentage of whey released, as well as whey composition, are presented (dry matter, crude protein and fat). Regarding acid gels, no differences on either released whey or whey composition could be linked to the presence of by-products in goat diets. If experiments 1 and 2 are compared, during Experiment 2 there was a trend showing that the released whey had higher contents of fat and protein, which is consistent with late lactation milk having less stability [24]. Released whey from goat milk gels in the present study was lower than that observed by Morgan et al. [16], which may be linked to differences in fat and protein content on milk from both studies, being highest in the present study, thereby leading to lower whey release.

Regarding the stability of rennet coagulated gels, no differences where attributable neither to diet nor to lactation stage. All diet groups released similar amounts of whey, and whey composition was similar for all groups. These results are consistent with those obtained for milk composition, as rennet coagulum properties are mainly dependent on protein, fat and mineral composition, which were similar for all diet groups [16,24,25]. Previous studies comparing grazing to fed with concentrates in dairy goats reported that such diet systems did not lead to significant differences in rennet coagulation aptitude [26]. Other factors may affect rennet coagulation ability; however, it should be taken into consideration that other effects such as pH, temperature and handling were kept constant in the present study. 

### 3.3. Milk Color 

Results of color parameters for experiment 1 and 2 are presented in Table 7. The inclusion of outer bracts and plant artichoke silages up to 12.5% inclusion in the diet of dairy goats did not affect milk color, while differences were observed for color parameters in the inclusion of 25%. In Experiment 2 (25% inclusion) L* increased, whereas b* and C* decreased in diets with artichoke, and these differences were small and could be due to pigments released during silage or to the small differences found in milk composition as the reduced crude protein and casein for the same experiment. Although these color differences may not be of practical value, since they less than 0.5 units, they were not visually perceptible.

Milk color depends on factors as the dispersion state of milk fat [27] and milk’s natural pigment concentration from carotenoids, proteins and riboflavin [28]. The dispersion state of milk fat has an important impact in physicochemical properties of cheese and butter [27]. L* depends on milk physical structure (dispersion of casein micelle and fat globules) that affect the diffusion of incident light (higher values of lightness are due to higher diffusion) [29]. Milk composition (fat, protein, Ca, P, etc.), technological treatments and measurement conditions affect the physical structure, and thus the L* value. In this study, the slightly lower protein and casein content in ABD-2 and APD-2 could be affecting the diffusion of light, increasing the L*. 

Coordinates a* and b* depend on natural pigment concentration of goat milk. Main pigments in milk are riboflavin (green compound), β-carotene (yellow coloration), and lutein, to a lesser extent [29]. Goats and sheep milk present a white color, because all of the β-carotene content is converted into retinol [27], and they are devoid of that pigment. The differences between diets in b* and C* could be due to small differences in natural pigments. The yellow coordinate (b*) and color saturation (C*) decreased in ABD-2 and APD-2, independently of whether the by-products were in the form of bracts or plant; they were higher in CD-2 group, therefore pointing to a replacement of ingredients from conventional feed that may have led to this reduction, however, it should be kept in mind that such differences are imperceptible to human eye, and so irrelevant from the organoleptic point of view. 

**Figure 1:**
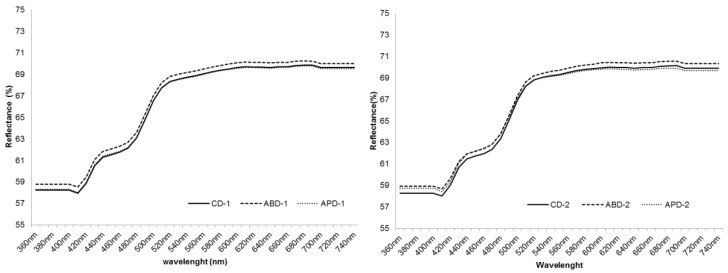
Reflectancies (360–740 nm) of milk from Murciano-Granadina goats fed on diets containing or not containing artichoke by-products (plant and outer bracts) in Experiment 1 (12.5% inclusion) and Experiment 2 (25% inclusion). CD-1: control diet in Experiment 1; ABD-1: artichoke bracts diet in Experiment 1; APD-1: artichoke plant diet in Experiment 1; CD-2: control diet in Experiment 2; ABD-2: artichoke bracts diet in Experiment 2; APD-2: artichoke plant diet in Experiment 2.

Figure 1 presents the reflectancies (360–740 nm) of milk from diets containing artichoke by-products (plant and outer bracts) with a 12.5% inclusion (Experiment 1) and 25% inclusion (Experiment 2). For both experiments the milk of goats fed with artichoke bracts (ABD-1 and ABD-2) showed higher values at all studied wavelengths. Several authors reported that using the 450–510 nm range from the analysis of milk, it is possible to explain 47–69% of the variability of milk β-carotenes, [29]. Differences observed in the present study are small, however, more studies are needed to find new relationships between the color of milk and its pigments, and the inclusion of higher amounts of silages from artichoke by-products. 

### 3.4. Milk Sensory Analyses

The sensory analysis of milk did not reveal the presence of off-flavors on any type of milk, and panelists were not able to identify milk sensory characteristics unique or related to any of the evaluated diets. Triangular tests were run by consumers; these are discriminant tests aiming to prove whether consumers were able to detect differences among sweetened yogurts prepared with milk from CD-2, ABD-2 and APD-2. As a result, only in 35% of the total number of triangular test consumers were able to provide correct judgements, and so, the minimum numbers of correct judgments needed to establish significance at 95–99% probability level (45–49) were not reached [17]. In detail: 34% of correct judgements were obtained when CD-2 vs. ABD-2 yogurts were compared; 30% when CD-2 vs. APD-2 yogurts were compared, and; 39% of correct judgements when ABD-2 were compared versus APD-2 yogurts. Such results point to a random selection of the different sample; consumers also commented how difficult was to discriminate among samples. In a similar trend, studies including up to 20% banana silages in goats diet lead to cheeses without flavor defects that kept sensory attributes similar to those of control cheeses [30]. The inclusion of buriti fruit oil in goats’ diet up to 4.5% dry matter did not modify milk sensory properties [31], and neither the inclusion of five cacti varieties [32]. Milk obtained from goats fed with up to 25% of silages of artichoke by-products did not develop off-flavors, and yielded yogurts of similar sensory properties that consumers were not able to discriminate. 

## 4. Conclusions

Silages from artichoke by-products (plant and outer bracts) can be successfully included in dairy goat balanced diets up to a 25% replacement of conventional ingredients without leading to relevant changes on milk composition, nor changes on technological properties of milk. The fact that changes in overall milk composition are irrelevant is the main factor preserving milk technological properties. It is of special relevance to point that there were no changes in the sensory properties of milk and yogurt obtained from those milks due to the inclusion of artichoke by-product silages. Further studies are needed to explore higher inclusion of preserved artichoke by products, as well as other horticultural by products in order to provide the scientific basis for their incorporation in balanced diets overcoming their seasonality, reducing feeding costs and enhancing farming sustainability.

## Figures and Tables

**Table 1:** Timeline. First row indicates the week since by-products were included in the diet: grey zone defines adaptation periods without milk sampling, white zones defines weeks when samplings were conducted.

1	2	3	4	5	6	7	8	9	10	11	12	13	14	15	16	17	18
		**Experiment 1**									**Experiment 2**						

**Table 2:** Chemical composition (% of DM) of diets containing or not containing artichoke by-products (plant and outer bracts) in the Experiment 1 (12.5% inclusion) and Experiment 2 (25% inclusion).

	Diets					
	Experiment 1			Experiment 2		
Items (% in Dry Material)	CD-1	ABD-1	APD-1	CD-2	ABD-2	APD-2
Dry matter	89.36	59.96	70.64	89.36	45.18	59.22
Crude protein	16.01	16.02	16.02	16.01	16.01	16.00
Crude Fibre	19.62	19.15	18.67	19.62	19.22	17.83
Neutral detergent fibre	37.83	38.52	38.07	37.83	39.81	38.32
Acid detergent fibre	24.32	24.84	24.26	24.32	25.97	24.22
Acid detergent lignine	5.65	4.32	4.24	5.65	3.30	2.93
Ash	7.24	7.08	7.70	7.24	7.38	8.42
Ether extract	4.65	4.85	5.05	4.65	4.89	5.32
MFU (MFU/kg DM)	0.92	0.94	0.92	0.92	0.95	0.91

DM: dry matter; CD-1: control diet in experiment 1; ABD-1: artichoke bracts diet in experiment 1; APD-1: artichoke plant diet in experiment 1; CD-2: control diet in experiment 2; ABD-2: artichoke bracts diet in experiment 2; APD-2: artichoke plant diet in experiment 2.

**Table 3:** Milk composition (g/100 mL) of Murciano-Granadina goats fed on diets containing or not containing artichoke by-products (plant and outer bracts) in Experiment 1 (12.5% include) and Experiment 2 (25% include).

**Experiment 1 (12.5% Inclusion)**				
	Diet			
Compound	CD-1	ABD-1	APD-1	Effect (*p*)
Fat	5.78 ± 0.60	5.90 ± 0.53	5.85 ± 0.57	NS
Crude protein	3.98 ± 0.24	3.83 ± 0.21	3.90 ± 0.22	NS
Casein	3.45 ± 0.21	3.34 ± 0.80	3.39 ± 0.19	NS
Whey protein	0.53 ± 0.04	0.49 ± 0.03	0.51 ± 0.04	NS
Lactose	4.21 ± 0.04	4.24 ± 0.05	4.20 ± 0.04	NS
Total solid	14.77 ± 0.87	14.72 ± 0.74	14.75 ± 0.79	NS
Ash	0.46 ± 0.07	0.42 ± 0.07	0.46 ± 0.07	NS
**Experiment 2 (25% Inclusion)**				
	Diet			
Compound	CD-2	ABD-2	APD-2	Effect (*p*)
Fat	5.61 ± 0.55	5.81 ± 0.61	5.43 ± 0.59	NS
Crude protein	4.01 ± 0.06 ^b^	3.79 ± 0.06 ^a^	3.91 ± 0.05 ^a,b^	**
Casein	3.50 ± 0.06 ^b^	3.33 ± 0.06 ^a^	3.39 ± 0.05 ^a,b^	**
Whey protein	0.52 ± 0.02	0.47 ± 0.04	0.51 ± 0.03	NS
Lactose	4.31 ± 0.06	4.33 ± 0.04	4.29 ± 0.06	NS
Total solid	14.76 ± 0.47	14.69 ^±^ 0.59	14.40 ± 0.55	NS
Ash	0.47 ± 0.01 ^b^	0.44 ± 0.01 ^a^	0.44 ± 0.02 ^a^	*

NS = not significant; * *p* < 0.05; ** *p* < 0.01; Treatment means of the ANOVA test (values are the mean value of all samplings from the same experiment). ^a,b^ Different letters in the same line indicate significant statistical differences between diets. CD-1: control diet in Experiment 1; ABD-1: artichoke bracts diet in Experiment 1; APD-1: artichoke plant diet in Experiment 1; CD-2: control diet in Experiment 2; ABD-2: artichoke bracts diet in Experiment 2; APD-2: artichoke plant diet in Experiment 2.

**Table 4:** pH and Dornic acidity (°D) at 0, 5 and 24 h of incubation (acidification capacity test) and alcohol test of the milk from Murciano-Granadina goats fed on diets containing or not containing artichoke by-products (plant and outer bracts) in the Experiment 1 (12.5% inclusion) and Experiment 2 (25% inclusion).

**Experiment 1 (12.5% Inclusion)**					
		Diets			
		CD-1	ABD-1	APD-1	Effect (*p*)
A0h	pH	6.77 ± 0.15	6.78 ± 0.15	6.81 ± 0.14	NS
	°D	14.88 ± 1.03	14.81 ± 0.65	14.31 ± 1.36	NS
A5h	pH	5.85 ± 0.40	5.82 ± 0.37	5.65 ± 0.39	NS
	°D	39.50 ± 10.26	39.06 ± 9.15	38.25 ± 8.53	NS
A24h	pH	4.17 ± 0.12	4.19 ± 0.13	4.19 ± 0.14	NS
	°D	97.88 ± 4.33	100.44 ± 3.32	99.69 ± 3.51	NS
	ALCOHOL TEST	47.13 ± 1.53	47.63 ± 0.88	48.00 ± 1.69	NS
**Experiment 2 (25% Inclusion)**					
		Diets			
		CD-2	ABD-2	APD-2	Effect (*p*)
A0h	pH	6.83 ± 0.11	6.70 ± 0.31	6.67 ± 0.29	NS
	°D	15.75 ± 0.61	15.67 ± 0.41	15.17 ± 0.93	NS
A5h	pH	5.51 ± 0.78	5.47 ± 0.94	5.48 ± 0.80	NS
	°D	36.72 ± 13.32	36.82 ± 13.38	37.72 ± 12.22	NS
A24h	pH	4.21 ± 0.06	4.18 ± 0.04	4.19 ± 0.05	NS
	°D	91.75 ± 7.17	96.17 ± 2.11	98.08 ± 2.44	NS
	ALCOHOL TEST	46.33 ± 1.03	46.67 ± 1.37	47.00 ± 1.79	NS

CD-1: control diet in Experiment 1; ABD-1: artichoke bracts diet in Experiment 1; APD-1: artichoke plant diet in Experiment 1; CD-2: control diet in Experiment 2; ABD-2: artichoke bracts diet in Experiment 2; APD-2: artichoke plant diet in Experiment 2. NS = not significant.

**Table 5:** Whey drainage ability (%) of milk and whey composition (fat, crude protein and total solid) for direct acidification from Murciano-Granadina goats fed on diets containing or not containing artichoke by-products (plant and outer bracts) in Experiment 1 (12.5% inclusion) and Experiment 2 (25% inclusion).

**Experiment 1 (12.5% Inclusion)**				
	Diets			
	CD-1	ABD-1	APD-1	Effect (*p*)
Whey drainage ability (%)	64.51 ± 3.23	65.23 ± 4.41	65.69 ± 2.92	NS
Fat (g/100 mL)	0.56 ± 0.14	0.56 ± 0.16	0.63 ± 0.20	NS
Crude protein (g/100 mL)	1.00 ± 0.06	1.02 ± 0.05	1.08 ± 0.05	NS
Total solid (g/100 mL)	8.27 ± 0.31	8.31 ± 0.42	8.41 ± 0.29	NS
**Experiment 2 (25% Inclusion)**				
	Diets			
	CD-2	ABD-2	APD-2	Effect (*p*)
Whey drainage ability (%)	63.69± 5.97	65.29 ± 7.27	68.29±3.14	NS
Fat (g/100 mL)	0.52 ± 0.21	0.50 ± 0.20	0.50 ± 0.20	NS
Crude protein (g/100 mL)	1.19 ± 0.46	1.28 ± 0.49	1.22 ± 0.32	NS
Total solid (g/100 mL)	8.64 ± 0.49	8.67 ± 0.42	8.52 ± 0.31	NS

CD-1: control diet in Experiment 1; ABD-1: artichoke bracts diet in Experiment 1; APD-1: artichoke plant diet in Experiment 1; CD-2: control diet in Experiment 2; ABD-2: artichoke bracts diet in Experiment 2; APD-2: artichoke plant diet in Experiment 2. NS = not significant.

**Table 6:** Whey drainage ability (%) of milk and whey composition (fat, crude protein and total solid) for rennet coagulated gels from Murciano-Granadina goats fed on diets containing or not containing artichoke by-products (plant and outer bracts) in Experiment 1 (12.5% inclusion) and Experiment 2 (25% inclusion).

**Experiment 1 (12.5% Inclusion)**				
	Diets			
	CD-1	ABD-1	APD-1	Effect (*p*)
Whey drainage ability (%)	64.32 ± 6.15	58.64 ± 6.17	59.86 ± 7.98	NS
Fat (g/100 mL)	0.19 ± 0.07	0.15 ± 0.06	0.18 ± 0.07	NS
Crude protein (g/100 mL)	0.91 ± 0.01	0.99 ± 0.04	0.99 ± 0.02	NS
Total solid (g/100 mL)	7.25 ± 0.08	7.22 ± 0.02	7.25 ± 0.10	NS
**Experiment 2 (25% Inclusion)**				
	Diets			
	CD-1	ABD-2	APD-2	Effect (*p*)
Whey drainage ability (%)	57.96 ± 5.66	55.48 ± 9.80	60.52 ± 4.28	NS
Fat (g/100 mL)	0.14 ± 0.01	0.29 ± 0.14	0.23 ± 0.02	NS
Crude protein (g/100 mL)	0.95 ± 0.06	0.98 ± 0.06	1.02 ± 0.07	NS
Total solid (g/100 mL)	7.26 ± 0.09	7.43 ± 0.17	7.34 ± 0.08	NS

CD-1: control diet in Experiment 1; ABD-1: artichoke bracts diet in Experiment 1; APD-1: artichoke plant diet in Experiment 1; CD-2: control diet in Experiment 2; ABD-2: artichoke bracts diet in Experiment 2; APD-2: artichoke plant diet in Experiment 2. NS = not significant.

**Table 7:** Color properties (CIELAB) of milk from Murciano-Granadina goats fed on diets containing or not containing artichoke by-products (plant and outer bracts) in Experiment 1 (12.5% inclusion) and Experiment 2 (25% inclusion).

**Experiment 1 (12.5% Inclusion)**				
	Diets			
	CD-1	ABD-1	APD-1	Effect (*p*)
L*	87.90 ± 0.49	87.56 ± 0.89	87.57 ± 0.81	NS
a*	−1.04 ± 0.14	−1.05 ± 0.14	−1.04 ± 0.16	NS
b*	4.89 ± 0.48	4.79 ± 0.72	4.91 ± 0.57	NS
C*	5.00 ± 0.50	4.91 ± 0.72	5.03 ± 0.58	NS
H*	102.04 ± 1.16	102.50 ± 1.29	101.95 ± 1.34	NS
**Experiment 2 (25% Inclusion)**				
	Diets			
	CD-2	ABD-2	APD-2	Effect (*p*)
L*	85.68 ± 1.09 ^a^	87.32 ± 1.00 ^b^	87.20 ± 1.08 ^b^	*
a*	−1.40 ± 0.12	−1.18 ± 0.10	−1.17 ± 0.17	NS
b*	6.10 ± 0.14 ^b^	5.63 ± 0.29 ^a^	5.68 ± 0.26 ^a^	*
C*	6.25 ± 0.53 ^b^	5.75 ± 0.29 ^a^	5.80 ± 0.25 ^a^	*
H*	102.90 ± 1.3	101.8 ± 0.81	101.7 ± 1.83	NS

CD-1: control diet in Experiment 1; ABD-1: artichoke bracts diet in Experiment 1; APD-1: artichoke plant diet in Experiment 1; CD-2: control diet in Experiment 2; ABD-2: artichoke bracts diet in Experiment 2; APD-2: artichoke plant diet in Experiment 2; NS = not significant; * *p* < 0.05); ^a,b^ Different letters in the same line indicate significant statistical differences between diets (*p* < 0.05).
